# A mixed methods analysis of existing assessment and evaluation tools (AETs) for mental health applications

**DOI:** 10.3389/fpubh.2024.1196491

**Published:** 2024-05-07

**Authors:** Sarah Ahmed, Chris Trimmer, Wishah Khan, Andrew Tuck, Terri Rodak, Branka Agic, Kelsey Kavic, Sapna Wadhawan, Maureen Abbott, M. Omair Husain, M. Ishrat Husain, Kwame McKenzie, Yuri Quintana, Farooq Naeem

**Affiliations:** ^1^Centre for Addiction and Mental Health (CAMH), Toronto, ON, Canada; ^2^Department of Psychiatry, University of Toronto, Toronto, ON, Canada; ^3^Mental Health Commission of Canada, Ottawa, ON, Canada; ^4^Harvard Medical School, Harvard University, Boston, MA, United States; ^5^Division of Clinical Informatics, Beth Israel Deaconess Medical Center, Boston, MA, United States

**Keywords:** mobile apps, mental health, digital health, guidelines, evaluation

## Abstract

**Introduction:**

Mental health Applications (MH Apps) can potentially improve access to high-quality mental health care. However, the recent rapid expansion of MH Apps has created growing concern regarding their safety and effectiveness, leading to the development of AETs (Assessment and Evaluation Tools) to help guide users. This article provides a critical, mixed methods analysis of existing AETs for MH Apps by reviewing the criteria used to evaluate MH Apps and assessing their effectiveness as evaluation tools.

**Methods:**

To identify relevant AETs, gray and scholarly literature were located through stakeholder consultation, Internet searching via Google and a literature search of bibliographic databases Medline, APA PsycInfo, and LISTA. Materials in English that provided a tool or method to evaluate MH Apps and were published from January 1, 2000, to January 26, 2021 were considered for inclusion.

**Results:**

Thirteen relevant AETs targeted for MH Apps met the inclusion criteria. The qualitative analysis of AETs and their evaluation criteria revealed that despite purporting to focus on MH Apps, the included AETs did not contain criteria that made them more specific to MH Apps than general health applications. There appeared to be very little agreed-upon terminology in this field, and the focus of selection criteria in AETs is often IT-related, with a lesser focus on clinical issues, equity, and scientific evidence. The quality of AETs was quantitatively assessed using the AGREE II, a standardized tool for evaluating assessment guidelines. Three out of 13 AETs were deemed ‘recommended’ using the AGREE II.

**Discussion:**

There is a need for further improvements to existing AETs. To realize the full potential of MH Apps and reduce stakeholders’ concerns, AETs must be developed within the current laws and governmental health policies, be specific to mental health, be feasible to implement and be supported by rigorous research methodology, medical education, and public awareness.

## Introduction

The COVID-19 pandemic has created numerous mental health challenges for the global population, including uncertainty, stress, and isolation (1, 2). Social distancing and changes in practice around COVID-19 have forced healthcare providers worldwide to provide their services through online platforms, thus acting as a catalyst to raise awareness, interest, and uptake of mobile Health Applications (mHealth Apps) (3). mHealth Apps are software applications on mobile devices that process health-related data and can be used to maintain, improve, or manage an individual’s health (4). Currently, the demand for mHealth Apps is high. A 2010 public survey found that 76% of 525 respondents would be interested in using their mobile phones for self-management and self-monitoring of mental health if the service were free (5). In a similar survey of physicians’ attitudes toward mobile health (mHealth), most expressed hope that technology could be very effective in their clinical practice (6). Recently, some countries have introduced legislation and policies to promote telemedicine by easing restrictions before the COVID-19 pandemic (7, 8). These changes varied across the countries, ranging from a relaxation of regulations due to the pandemic and easing of restrictions on prescription medications, to telepsychiatry services being reimbursed at the same rate (or higher) than in-person consultations during the COVID-19 pandemic. However, no follow-up data is available on the current state of these changes and their impact (8).

The IQVIA Institute for Human Data Science estimated that more than 318,000 Health Apps were available in 2017 (9), with more than 10,000 Apps explicitly designed for mental or behavioral health (10). With the number of available mHealth Apps on the rise, so are the concerns regarding their effectiveness and safety. Given the rigorous assessment pharmaceuticals and medical devices must undergo to be licensed, there is an increasing call to apply the same rigor for mHealth Apps to ensure safe and effective implementation of state-of-the-art technology into healthcare (9). This is especially important for Mental health Applications (MH Apps), which hold the potential to improve access to high-quality mental health care.

There is insufficient evidence for the effectiveness of MH Apps, with one paper reporting that only 3.4% of MH Apps were included in research studies to justify their claims of effectiveness, with most of that research undertaken by those involved in developing the App (11). A team of researchers reviewed seven meta-analyses of MH Apps for the quality of available evidence with respect to the use of mental health applications and found that the studies were generally of lower quality and did not offer strong empirical support for the effectiveness of the Apps (12). The problem is further compounded by the observation that randomized controlled trials (RCTs) in this area rarely report the details of the MH App they are providing to research participants (13). Therefore, in order to improve the effectiveness of MH Apps, high-quality, evidence-based research must be conducted to evaluate them. This will allow for the development of standardized guidelines that can be used widely to objectively and regularly assess existing and future MH Apps.

Evidence-based guidelines that have been developed for mental health interventions (e.g., National Institute of Clinical Excellence in England and the APA in the United States) have generally not been applied to MH Apps, likely due to the significant differences in delivery mediums. Only minimal guidance is available on (a) the development and reporting of MH Apps, (b) their effects and side effects, (c) information on matters related to privacy and security, and (d) their scientific testing and reporting (14). Notably, the demand for mobile health App guidance and regulation has increased (15). The National Health Service (NHS) in England, for example, developed an *Apps Library*, which publishes lists of health applications reviewed using a standard set of criteria, including security and clinical safety, outcomes, value for money, focus on user needs, stability and simplicity of use and evidence base (16). The United States of America’s Food and Drug Administration (FDA) provides regulatory oversight on Apps that function as medical devices and may pose risks to patients (17). Similarly, the European Commission (EC) has issued its own guidelines for app developers (18). In Germany, the DiGA (Digitale Gesundheitsanwendung or Digital Health Applications in English) is a set of health legislation and rules aimed allow digital healthcare applications to be prescribed by doctors, similar to the way medications are prescribed, for a variety of diagnoses including mental health conditions (19).

Clinicians, healthcare providers, policymakers, and members of the general public have identified a need for more specificity and coordination in making an informed decision when selecting an MH App (20). Care providers need more information on the skills and knowledge required to convey timely information and recommend safe and effective app use (21, 22). This need has led to the development of AETs (Assessment and Evaluation Tools) to help guide users. AETs can include frameworks, guidelines, rating systems, or App libraries that assess and/or evaluate a mobile health application, including MH Apps, for various criteria, such as privacy, clinical information, user experience and authenticity. This paper aims to provide a better understanding of the existing AETs for MH Apps and provide insights for service providers and for people with lived experiences with mental health problems. For health professionals, a better understanding of AETs can lead to the development of easy-to-use and evidence-based “prescribing guidelines.” For MH App users, a greater understanding of AETs could ultimately result in easy-to-read product information regarding side effects, and relevant privacy, security, and quality issues. It is, therefore, important that AETs provide guidance to professionals as well as the general public in a manner that is easily understandable, such as providing both technical reports and lay-person summaries.

A literature review and qualitative analysis of existing assessment and evaluation tools for MH Apps was conducted to understand the existing standards and guidelines. To assess the strengths and limitations of existing AETs for MH Apps, the overall quality of AETs was quantitatively analyzed using the Appraisal of Guidelines for REsearch and Evaluation, version 2 (AGREE II). The AGREE II is a commonly used instrument to evaluate guidelines that identify best practices in guideline or framework development (23).

### Objectives

The primary objective of this study was a qualitative analysis of evaluation criteria of AETs and identifying the strengths and limitations of these tools. The secondary objective was to assess the existing AETs quantitatively against existing standards using the AGREE II tool.

## Methods

We began with a synthesis of existing AETs using a broad scan of literature in the field in order to: (a) understand the context of AETs (e.g., information on AET developers, types of Apps to evaluate and intended user audience) (b) collect information on criteria used for evaluation and (c) identify resources, links, and gaps. In addition to Internet and literature searches (including a bibliography scan of available tools), we connected with knowledgeable stakeholders recommended by experts in the field through personal and professional networks. These stakeholders were mental health app developers (*n* = 2), mental health professionals (*n* = 3), mental health professionals with specific interest in evaluation and implementation of MH Apps (*n* = 6), framework developers (*n* = 3), mental health leaders (e.g., Chief or head of department; *n* = 3), mental health app user (*n* = 1), mental health policy makers (e.g., individuals who work with the government; *n* = 3) and mental health educators (*n* = 2) across Canada and abroad. A list of national and international stakeholders was constructed, and they guided an initial list of AETs.

We then conducted a narrative literature review (24) of AETs for mHealth and MH Apps and related publications. We reviewed AETs for both mobile health and mental health applications to encompass all available AETs for MH Apps. The following are the methods and results of the literature review.

## Literature review

### Search strategy

We identified AETs for mHealth and MH Apps using a three-pronged approach: (a) gathering tools via stakeholder feedback (providing recommendations of AETs to include in our review) and internet searching (Google and Google Scholar) (b) a focused literature search using bibliographic databases, and (c) a focused search of peer reviewed publications in this area.

**Figure 1:**
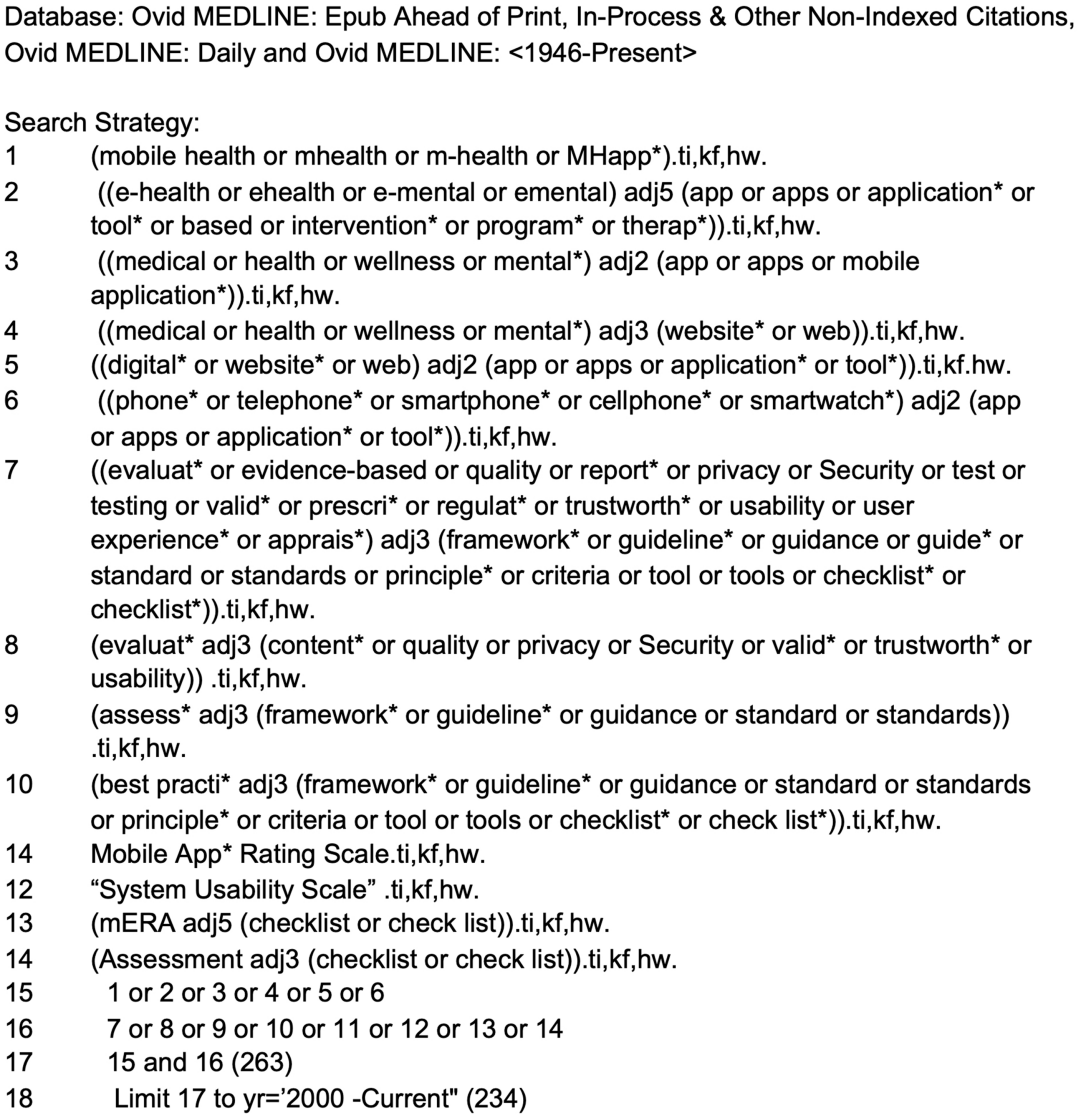
MEDLINE search strategy used for the literature review.

The literature search for scholarly articles was conducted by a health sciences librarian (TR) who developed the search strategy with input from the research team. The strategy used database-specific subject headings and keywords in the following databases: Medline (including Epub ahead of print, in-process, and other non-indexed citations), APA PsycInfo, and Library, Information Science and Technology Abstracts (LISTA). The search strategy included terms for mobile and e-health applications (e.g., mobile health, mhealth, digital tools), terms for mental health applications (e.g., mental, e-mental, wellness) combined with terms for evaluative frameworks (e.g., evaluation, usability, best practice framework, guideline, standards), as well as names of commonly used frameworks already known to the research team. As they arose in the results, app rating scales were also included in the search if they were a part of a framework. The year range was from January 1, 2000 to January 26, 2021 (the date of search execution). The strategies were designed to favor specificity over sensitivity, as this was not intended to be a comprehensive systematic or scoping review. See Figure 1 for the MEDLINE search strategy.

### Inclusion and exclusion criteria

Though not a systematic review, we engaged in a formal screening process using eligibility criteria to streamline our selection process. The inclusion criteria for the literature review were studies in English that provided a tool or method to evaluate MH Apps and were published from January 1, 2000, to January, 2021. Studies in a language other than English and studies on mobile applications unrelated to a mental health area were excluded.

### Data extraction

The following data points were collected from each paper: author, organization affiliation, year of publication, name of the AET, country of origin, description of the framework, and the evaluation criteria of the AET.

### Study selection

Once the duplicates (including multiple papers reporting on the same AET used in a different research context) had been removed, two researchers (CT and WK) reviewed the document titles and abstracts independently. Finally, three researchers (FN, CT, and WK) met to agree on the final list of documents. Titles unrelated to the topic, scientific and popular articles, news articles, books, presentations, and opinion pieces unrelated to AETs were excluded. Each researcher evaluated the documents against the inclusion criteria and screened the document’s reference list for additional resources. Independent results were compared between the two researchers (CT and WK). When discrepancies existed, a third researcher (FN) was involved in resolving eligibility disagreements.

### Methods of analyses

1. Qualitative analysis of AET criteria.

We used the constant comparative method (CCM) to analyze the qualitative data and determination of themes (25, 26). This qualitative analysis method combines inductive coding with a simultaneous comparison of all attributes obtained from our data (26). Researchers applied open coding as a first step in the coding process (CT and WK) to identify attributes and allow categories of AET evaluation criteria to emerge from the data. In open coding and comparison, initial categories were changed, merged, and omitted when necessary. The second step involved axial coding to explore connections between categories and sub-categories. Selective coding as a third step involved selecting the core themes of AET evaluation. To better understand the technological terminology of the AETs, we consulted team members with expertise in Information Technology (IT).

1. Quantitative analysis: quality assessment of AETs using AGREE II Tool.

We used the AGREE II scale to assess the quality, methodological rigor, and transparency of each AET (23). The AGREE II provides an overall score to assess the methodological quality of guidelines and provide a level of recommendation (strongly recommend, weakly recommend or recommend) of use for clinical practitioners. The AGREE II includes the following domains to guide assessment of AETs: *Scope and Purpose* (i.e., the overall aim of the guideline, the specific health questions, and the target population); *Stakeholder Involvement* (i.e., the extent to which the guideline was developed by the appropriate stakeholders and represented the views of its intended users); *Rigor of Development* (i.e., the process used to gather and synthesize the evidence, the methods to formulate the recommendations and to update them); *Clarity of Presentation* (the language, structure, and format of the guideline); *Applicability* (the likely barriers and facilitators to implementation, strategies to improve uptake, and resource implications of applying the guideline); and *Editorial Independence* (the formulation of recommendations not being unduly biased with competing interests).

Twenty-three key items across six domains were scored on a Likert scale from one to seven, with one being strongly disagree and seven being strongly agree. The score for each domain was obtained by summing all scores of the individual items in each domain and then standardizing as follows: (obtained score - minimal possible score)/(maximal possible score - minimal possible score) (27, 28). While the AGREE II instrument does not provide a universal standard on how to interpret scores, we used commonly described criteria (27, 28) for overall assessment and recommendation of AET quality: *strongly recommended* if five to six principal domain scores were ≥ 50%; *recommended* if three to four domain scores were ≥ 50%; *weakly recommended* if one to two domain scores were ≥ 50%, and *not recommended* if all scores were below 50%.

## Results

**Figure 2:**
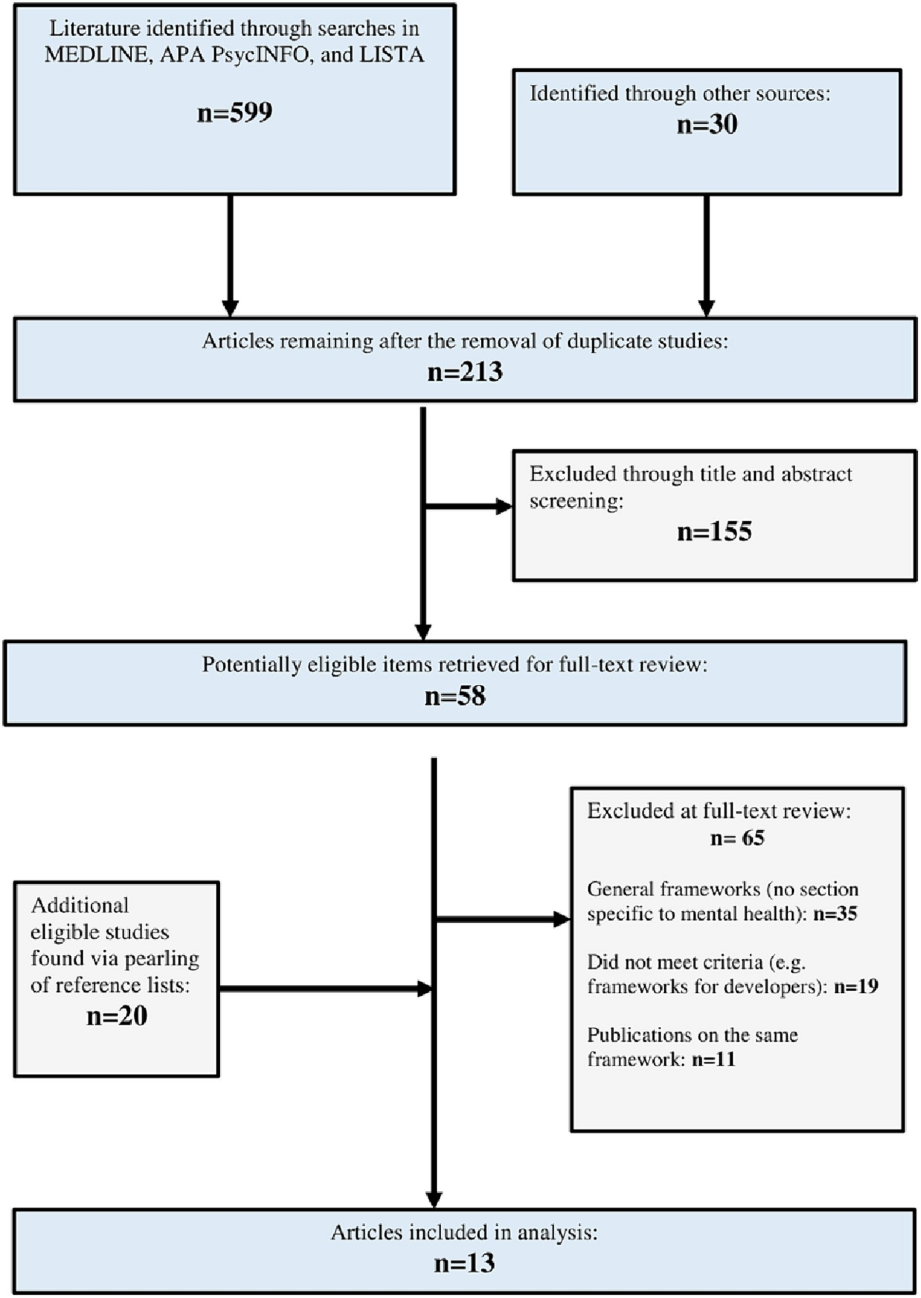
Overview of study selection process.

Our three-pronged search identified 599 citations of potentially relevant titles and abstracts from the academic research literature. An additional 30 literature sources were identified through other search methods (including a Google and Google Scholar literature search). Duplicate, non-applicable, and redundant records were removed, with 213 records remaining. A total of 155 literature sources were then excluded as they did not meet the inclusion criteria. The remaining papers (*n* = 58) were deemed eligible for inclusion based on their relevance to an AET. An additional 20 papers were deemed eligible from a review of reference lists (*n* = 78). Following a full-text review of these items, 65 items were excluded for the following reasons: 35 papers described general health AETs, 19 papers did not describe frameworks or guidelines that met the criteria of an AET, and 11 discussed AETs already identified in other included articles. Hence, 13 AETs (15, 21, 22, 29–38) met the inclusion criteria. See Figure 2 for an overview of the study selection process.

### Overview of AETs

Table 1 describes the overall characteristics of the AETs. Of the 13 selected AETs, six (46%) were developed in Canada (21, 22, 29–32), five (38%) in the United States (33–37), one (8%) in England (15) and one (8%) in New Zealand (38). Five (38%) AETs were developed by non-profit organizations (22, 34–36, 38), two (15%) by national professional organizations (33, 37), one by a local health service (29), another by a national health service (15), two by hospitals (31, 32), one by a national non-profit organization created by the government (30), and one by an individual as a Ph.D. (21) project. Three (23%) tools focused on general health Apps with dedicated sections on mental health (15, 36, 38), and the rest (77%) of the tools focused solely on MH Apps (21, 22, 29–35, 37). Three tools used the term *frameworks* (22, 30, 37), one used *app directory* (29), one used the term *app library* (31), and one used *app rating index* (21). The rest (54%) (15, 32–36, 38) were online libraries (i.e., websites) without a specific term to represent the AET.

**Table 1:** Description of assessment and evaluation tools (AETs) for MH apps.

	Source affiliation	Country of origin	Intended audience	Area^a^	Type of eval. criteria^b^	Dev. details^c^	Type of tool	Implemented	Policy on update	Stakeholder engagement
Alberta Health Services (29)	Alberta Provincial Health Services	Canada	Clinicians, Researchers, App Developers	General	Selection criteria	No	App Directory	No	None	No
Strudwick (32)	CAMH (Hospital)	Canada	Health Care Providers (HCPs)	General	Questionnaire	Yes	Digital resources	No	None	No
Azad-Khanegah (21)	Individual PhD	Canada	HCPs, General Public	General	Rating scale	Yes	App rating index	No	None	Yes
MHCC (30)	Mental Health Commission of Canada (MHCC)	Canada	HCPs, Patients, App Developers	General	Selection criteria	Yes	Framework	Implement kit, but not implemented	None	Yes
Homewood (22)	Homewood (Non-profit organization)	Canada	HCPs	Youth	Selection criteria	Yes	Framework	No	None	Yes, limited details
Scarborough Health Network (31)	Scarborough Health Network	Canada	Patients	General	N/A	No	Mental Health App Library only	No	None	No
ADAA (33)	Anxiety and Depression Association of America (ADAA)	USA	Patients	General	Ratings key	No	Online library, Reviews	No	None	No
MindTools (34)	MindTools (Non-profit Organization)	USA	Patients and Clinicians	General	Rating scale	No	Online library	Yes, website	None	No
NHS, UK (15)	National Health Services (NHS) England	England	Patients, Service Providers	General	Selection criteria	No	Online library	Yes, website	None	No
One Mind (35)	Non-profit organization	USA	Professionals, Researchers, Patients	General	Rating scale	No	Online Library	Yes, website	None	No
Ranked Health (36)	Ranked Health (Non-profit organization)	USA	Providers and Patients	General	Rating scale	Yes	Online Library	Yes, website	None	No
APA (37)	American Psychiatric Association (APA)	USA	Clinicians and Patients	General	Hierarchical Selection criteria	Yes	Framework, Online Library	Yes, website	None	Yes, limited details
Health Navigator (38)	Non-profit organization	New Zealand	Patients	General	Selection criteria	No	Online Library	Yes, website	None	No

^a^Problem area and population. ^b^Type of evaluation criteria. ^c^Details of development.

These AETs used a variety of methods to assess app quality. Four (31%) AETs used rating scales (21, 34–36), one (8%) used a rating key (33), and another provided a questionnaire (32) to assess MH Apps. The rest (54%) of the tools used pre-selected criteria from which to assess app quality (15, 22, 29–31, 38). One tool offered a hierarchical selection criterion (37). Another AET assessed Apps in four stages: (a) internal review, (b) relevance to sponsoring country review, (c) clinical review, and (d) user review (38). Only two tools guided readers on how to use the selection criteria (22, 37). None of the AETs provided details on how the framework would be updated in the future (i.e., an updated policy).

We were able to find details on how these tools were developed (methodology) for only six (46%) of the AETs (21, 22, 30, 32, 36, 37). Limited information on stakeholder engagement in these AETs was available, with a noticeable absence of app distributors, app developers, and health funders. Even when an AET claimed to engage all stakeholders, little or no information was available on how these stakeholders were engaged. In terms of implementation, one of the AETs was associated with an implementation toolkit (30), and another AET is being used to guide an app-evaluating website (37).^1^ Six AETs (46%) are a part of websites (15, 33–36, 38) that provide online guidance on applications using various selection criteria. No information on implementation was available for the remaining five (38%) AETs (21, 22, 29, 31, 32). Apart from the NHS *App Library* (15), none of these tools have been adopted by a health system at a national level. No information is available on the evaluation of their implementation. No data is available on how useful these AETs are in helping healthcare professionals and clients make informed choices. None of the AETs specified the population except one focused on youth (22). None of the AETs specified the problem areas (e.g., general well-being or a specific disorder). Similarly, no data is available on the number of MH App downloads or how these Apps are used.

The AETs in this environmental scan were included based on their stated focus on assessing and evaluating MH Apps. However, during analysis, our research team noted that these AETs are relatively non-specific to mental health issues and could be used as assessment and evaluation tools for general health applications. This observation has also been acknowledged by two of the AET developers (22, 37).

1. Qualitative analysis of app assessment and evaluation criteria.

The research team (FN, WK, and CT) listed, then grouped, common themes across AETs to determine broad categories of AET criteria. Qualitative analysis of the 13 included AETs revealed seven themes: (a) *Authenticity of Content, Source and Process* (whether experts developed the content, whether users were involved in the development process and the app developer’s background); (b) *Ethical and Legal Issues* (issues related to privacy and security, data sharing and data security); (c) *User Experience and User Engagement* (issues related to usability, user desirability, functionality, user engagement, customization, and personalization); (d) *Cost* (how much the app costs, in-app purchases); (e) *Clinical Use and Indications* (whether there are clearly described clinical indications); (f) *Risk to User* (whether there is a potential of harm caused by the App to the user); (g) *Technology-Related Issues* (whether the App provides technical information, and whether the app user has access to necessary equipment); and (h) *Evidence* (both scientific evidence and the number of downloads). Table 2 displays an overview of AETs assessed for the criteria mentioned above. At the same time, the themes often overlapped and a clear distinction between themes was not possible. Various sub-categories were identified and described under the major themes. These themes and sub-categories are displayed in Table 3 to indicate the variation and similarities of themes discussed in the 13 AETs.

**Table 2:** Qualitative analysis of evaluation criteria for each assessment and evaluation tool.

	Authenticity-content, source & process	Evidence	User experience	Cost of the apps	Clinical use and indications	Risk to user	Ethical and legal	User engagement	Technology-related issues
Alberta Health Services (29)	Y	Y	N	N	N	N	N	N	N
Strudwick (32)	Y	Y	N	Y	Y	Y	Y	N	Y
Azad-Khanegah (21)	Y	Y	Y	Y	Y	Y	Y	N	N
MHCC (30)	Y	Y	Y	Y	Y	N	Y	Y	Y
Homewood (22)	Y	Y	Y	N	Y	Y	Y	Y	Y
Scarborough Health Network (31)	N	N	N	N	N	N	N	N	N
ADAA (33)	N	Y	Y	N	N	N	N	N	N
MindTools (34)	Y	Y	Y	N	N	N	Y	Y	Y
NHS, UK (15)	N	N	Y	N	N	Y	Y	N	Y
One Mind (35)	Y	Y	Y	N	Y	N	Y	N	N
Ranked Health (36)	Y	Y	Y	N	Y	N	Y	N	N
APA (37)	Y	Y	Y	Y	Y	Y	Y	N	Y
Health Navigator (38)	Y	N	Y	Y	N	N	Y	Y	Y

Y, yes, included; N, no, not included.

**Table 3:** Summary of themes and criteria assessed by 13 assessment and evaluative tools.

	Themes and criteria
Alberta health services (29)	Expert opinion, Evidence (evidence from research), Source reliability (reliable developers)
Strudwick (32)	Ethical and legal (level of the consent), privacy, security and confidentiality, Risks (unintended consequences), Clinical use (benefits), reliability (accurate, and trustworthy), evidence (effective), Digital literacy (user skills in technology use), Access to technology, Cultural issues (Language barriers), Cost
Azad-Khanegah (21)	User interface (Esthetics), Cost (Affordability), Customizability, User experience (Ease of use, User engagement, Functionality,), Privacy & Security, Reliability (Trustworthiness), Clinical use (Usefulness) Evidence (link to scientific studies)
MHCC (30)	Evidence (effectiveness), Ethical and legal (Transparency of Information), Security, Information Security, User experience (Functionality, Usability), Source Reliability (Developer Transparency, Funding Transparency), User involvement in app development (user Inclusion, User Desirability, Meaningful Inclusion), Clinical use (Audience), Technical info (Supported Platforms, Interoperability), Cost (App Price)
Homewood (22)	Clinical use (Intended use and users), Source reliability (legal owner, funding), cost, Validity (Content review, face validity), Technical information (update cycle), User engagement (user input), behavioral model, User experience (prototype usability, Usability testing) Personalization, Legal and ethical (user consent, ethical principles, user data ownership and control, data sharing), Security & privacy, Technical information (technical requirements, interoperability), user engagement (user engagement, user feedback), Risk (no harm), Evidence (efficacy and dose-effect, effect size, effect over time, factor analysis, bias, sensitivity analysis, reproducibility)
Scarborough health network (31)	No criteria
ADAA (33)	User experience (Ease of Use, Interactive/feedback), Evidence (Effectiveness, Research), Personalization, Source reliability (Developer Identity, who are the developers?), Privacy (How private is your phone?), Validity (Content: What do MH apps claim to do?), Clinical use (Target Users-Who are the MH apps for?)
Mind tools (34)	User experience (usability, visual design, therapeutic alliance, strong advisory support,) User engagement (user engagement), Validity (content), Source Credibility (owner’s credibility), Technical info (maintenance/frequency of updates), Source reliability (third-party endorsement), evidence of successful implementation, Privacy & Security, Legal and ethical (confidentiality, explanation of data journey, how data is used)
NHS, UK (15)	Risk (Clinical Safety), Security and privacy (Data protection), Functionality (Technical assurance, secure & stable), Technical info (Interoperability), User experience (Usability), Access to technology (Accessibility)
One mind (35)	Credibility, Evidence (research evidence), Rigor of development, Clinical use (clarity of purpose), User Experience, Security & Privacy Practices (Transparency) (data security, privacy policy)
Ranked health (36)	Evidence (Effectiveness, evidence-based) Clinical use (clinical relevance), credibility, Functionality, features, Legal and ethical (data sharing), Technical info (integration with other apps or medical), User experience (Usability, user interface, user experience), Access to technology (accessibility), Privacy & Security, Validity (Clinical foundation), User experience (Engagement style), Clinical use (Therapeutic Goal)
APA (37)	Background info (Business model, Credibility, Medical claims, Technical Costs and advertising Stability) Privacy and security (Data collected Data storage Deleting personal data Personal health information Security measures in place Privacy policy) Evidence based (First impressions, Impression after using, Clinical validity, User feedback supporting) Ease of use (Specificity to users and accessibility Short-term usability Long-term usability) Data integration (Data ownership access and export Clinically actionable Therapeutic alliance)
Health navigator (38)	Source reliability (Credibility, content quality, source quality), privacy & security, User experience (interactivity, appearance, fun & entertaining, ease of use), User engagement (stakeholder involvement, inclusive), Cost (cost consideration), Cultural issues (language), Access to tech (accessibility)

### Authenticity of content, process and source

This theme includes three sub-categories (a) *Authenticity of Content* (whether experts developed the content); (b) *Authenticity of the Process* (were users involved in the development process); and (c) *Authenticity of Source* (the app developer’s background).

Ten (77%) (21, 22, 29, 30, 32, 34–38) AETs recommended *Authenticity of Content* or *Source* criteria. Of these, six (46%) (15, 21, 22, 30, 32, 37) considered the authenticity of the source (i.e., reliability of the app developer or third-party partnership). Four (31%) (15, 21, 22, 37) considered the authenticity of the content, most commonly using the term ‘validity’ (specifically, face validity) of the MH App content.

Most tools highlighted the importance of app developers’ credibility (e.g., the type of business model used, source of funding, and transparency). AET developers used a variety of parameters and terms to describe authenticity criteria. For example, one AET (30) describes the criterion *Source Reliability* as consisting of developer and funding transparency. This tool also discussed user involvement in app development that consists of *User Inclusion, User Desirability and the Meaningful Inclusion of Users*. Another tool (34) considers third-party endorsements and the owner’s credibility to be indicators of the source’s authenticity.

Only three (23%) tools mentioned content as a criterion for evaluation. Only one tool (8%) (22) considered the cognitive and behavioral model from which the mental health application is derived as a criterion.

### Ethical and legal issues

Nearly all the tools used ethical and legal standards as a criterion. Three sub-categories emerged under this theme: (a) *Privacy* (the safeguarding of user identity) and *Security* (the safeguarding of data); (b) *Data Management* (collecting, keeping, sharing, using or discarding data securely, efficiently, and cost-effectively); and (c) *Diversity and Equity* (diversity refers to the traits and characteristics that make people unique, while equity refers to providing everyone with the full range of opportunities and benefits).

*Privacy and security* concerns for the app user were included by 11 (85%) (15, 21, 22, 30, 32–38) of the tools. Of these, three (23%) (22, 30, 37) specified a specific assessment of whether a data collection policy was published, and two AETs (15%) (21, 22) assessed the extent of securing personal data collected. Ethical and legal concerns for the app user were assessed by seven (54%) (15, 21, 22, 32, 34, 35, 37) of the tools. Major app stores require a privacy policy before publishing an app (39). However, these policies have a broad focus. The complex legal language used in these policies might also make it difficult for people living with mental health problems and clinicians to comprehend the language.

Some of the AETs mentioned the need to consider user characteristics and *diversity, equity and cultural factors*. For example, one (30) AET explicitly highlighted the need for *gender responsiveness* (i.e., does the App consider the needs and preferences of men, women, boys, girls and gender-diverse people?). Two AETs highlighted the need for *cultural appropriateness* (i.e., how appropriate is the App for people from various cultures?) (22, 30). However, this emphasis did not reflect the focus audience or the selection criteria of our highlighted AETs. One AET (32) used language appropriateness as a selection criterion. Only one AET (22) included criteria that had special considerations for applying evaluation criteria for youth regarding privacy regulations, consent of minors, and personalization of content by age and culture. Two of the AETs (22, 33) used personalization as a selection criterion.

### User experience and user engagement

Nine (69%) (15, 21, 22, 30, 33–36, 38) AETs used user experience as a criterion. Four (31%) (22, 30, 34, 38) used engagement as a criterion. Six (46%) (21, 22, 30, 32, 34, 37) AETs proposed the functionality of the App as selection criteria. In comparison, four (22, 30, 34, 37) assessed the quality of the user interface of the App (including the esthetics and ease of use), and one (38) used the criteria of how fun or engaging the App was for the user. Finally, five (38%) (21, 22, 30, 32, 37) AETs include criteria to evaluate whether user engagement was included in the development and maintenance of Apps. The most important sub-categories to clinicians, researchers and clients might be “user engagement,” which is equivalent to “treatment adherence or compliance.”

### Evidence

Most AETs (21, 22, 29, 30, 32–37) considered evidence as a selection criterion using varied terminology and concepts. This theme can be divided into three categories: (a) *Empirical evidence*, (b) *Implementation Info*, and (c) *Cost-effectiveness*.

Ten (77%) (21, 22, 29, 30, 32–37) of the AETs suggested *evidence* as an app evaluation criterion. However, there is no consensus on what can be the evidence that an App is effective. While the terms evidence, evidence-based, and effectiveness were used by most (21, 22, 29, 30, 32–37) of these AETs, only one AET (22) described the concept in some detail. This AET proposed that evidence consists of efficacy and dose effect, effect size, the effect over time, factor analysis, bias, sensitivity analysis, and reproducibility. This AET also suggested how these parameters could be assessed. Another AET (37) considered a link to scientific studies as sufficient for evidence.

*Cost-effectiveness*, an essential parameter in selecting health interventions, can be understood as the trade-off between the MH App’s benefits and the App’s cost (e.g., to the individual, to the clinician, or the overall healthcare system). Potential indirect benefits include improved physical health, enhanced current and future productivity, and reduced caregivers’ demands (40). Currently, limited information is available on the cost-effectiveness of MH Apps. None of the AETs used cost-effectiveness as a selection criterion.

### Clinical use and indications

Seven (54%) (21, 22, 30, 32, 35–37) AETs used clear descriptions of clinical indicators as a selection criterion. One AET (30), for example, considered clinical claims and target users to be an indicator of clinical use criteria. Health Apps exist on a spectrum, from consumer-facing, non-regulated, non-interventional Apps like fitness trackers to regulated, prescription-only Apps like digital therapeutic to manage substance use disorder (41). A wide variety of MH Apps are launched under the “well-being” categories rather than with specific “clinical indications.” The issue becomes more complicated considering the legal applications; for example, it has been suggested that because most Apps are categorized as ‘health and wellness’ Apps, they are not designated as medical devices and thus fall outside the purview of the FDA guidelines. Those which may be medical Apps have utilized the regulatory discretion pathway to avoid scrutiny (42).

### Risk(s) to the app user

MH Apps have the potential to cause significant risks and as such, governmental guidelines take a risk-based approach to evaluating mhealth Apps. Risks to Users can be considered under two categories: (i) *technology-related risks* and (ii) *clinical risks*. Five (38%) (21, 22, 30, 32, 37) of the AETs considered the risk to the users (potential of harm caused by the App). All AETs, however, focus on *technology-related risks* such as risks due to privacy, security or data-related issues. There is considerable overlap of the first category with privacy and security and data management under *ethical and legal issues*. There is sufficient evidence to indicate that not all health Apps are safe; based on traffic, content, and network analysis of health Apps reported that 79% of sampled Apps shared user data (43).

The issue of *clinical risks* has not received attention in AETs. Only one AET uses the term clinical safety (i.e., Is the App assessed to ensure that baseline clinical safety measures are in place and that organizations undertake clinical risk management activities to manage this risk?). Clinical risks can be further considered as (a) risks due to inaccurate health-related information (44); (b) increased risk of harm to self or others due to the App use (21); (c) smartphone addiction (45); and most significantly, (d) side effects of interventions that provide psychotherapy (46).

### Cost of the apps

The cost of mental health services is a significant barrier to accessing care for people with mental health problems (47). The users must be aware of the *business model* to make an informed decision. Currently, health systems do not offer a system supporting the purchase of mhealth Apps. Only four of the AETs (31%) included the cost in their evaluation models. One AET assessed cost with a distinction between initial cost and ongoing (or in-app) purchases (32).

### Technology-related issues

Three categories were identified in this theme (i) *Digital literacy* (skills related to the effective and appropriate use of technology), (ii) *Access to technology* and (iii) *Access to technical Info*. Seven (54%) (15, 22, 30, 32, 34, 37, 38) AETs considered at least one aspect of technology-related issues as their selection criteria. However, only one (32) AET listed user skills as a criterion in app selection. Five (38%) (15, 22, 30, 32, 37) AETs assessed the App’s update cycle frequency, the degree of technology integration across platforms (including the number of supported platforms and interoperability), and minimum technical requirements for usage. Four (31%) (15, 21, 31, 36) AETs assessed issues of accessibility, with two AETs (30, 32) defining accessibility as the user’s access to technology or digital literacy, and two AETs (22, 30) assessed the MH App’s recognition of cultural issues for the user, such as a language barrier.

1. Quantitative analysis: quality assessment of AETs using AGREE II tool.

Table 4 displays the core scoring domains for each of the 13 AETs on the AGREE II. To assess the quality, methodological rigor, and transparency of each AET, we used the AGREE II scale, a standardized tool for evaluating guidelines (23). On examination of independent assessment domains using prevalent acceptable criteria of a score greater than 50% (27, 28), we found that: seven (54% of total) AETs met the criteria on the first domain, *Scope and Purpose* (15, 21, 22, 29, 30, 36, 37); three (23%) AETs met the criteria on the domain *Stakeholder Involvement* (21, 30, 37); four (31%) tools met the criteria for *Rigor of Development* (21, 30, 32, 37); seven (54%) AETs met the criteria for *Clarity of Presentation* (22, 29, 33–35, 37, 38); and none (0%) of the tools met the criteria for *Applicability or Editorial Independence*. Using the criteria of ‘number of domains with ≥50%’ for overall assessment and recommendations, only three (23%) AETs met the criteria for ‘recommended’ (21, 30, 37), and one (8%) met the criteria for ‘not recommended’ (31), the rest (69%) were all within the ‘weakly recommended’ category (15, 22, 29, 32–36, 38).

**Table 4:** Domain-scaled scores on AGREE II for each assessment and evaluation tool.

	Scope and purpose	Stakeholder involvement	Rigor of development	Clarity of presentation	Applicability	Editorial independence	Domains with >50%
Alberta Health Services (29)	66%	31%	15%	57%	6%	8%	2
Strudwick (32)	31%	33%	65%	33%	47%	22%	1
Azad-Khanegah (21)	96%	93%	74%	48%	46%	33%	3
MHCC (30)	89%	63%	65%	33%	47%	33%	3
Homewood (22)	96%	6%	45%	67%	26%	8%	2
Scarborough Health Network (31)	6%	15%	1%	6%	8%	0%	0
ADAA (33)	6%	17%	1%	59%	1%	11%	1
MindTools (34)	43%	43%	32%	63%	25%	0%	1
NHS, UK (15)	70%	25%	15%	11%	25%	0%	1
One Mind (35)	48%	19%	21%	52%	11%	8%	1
Ranked Health (36)	56%	37%	13%	19%	8%	0%	1
APA (37)	96%	74%	64%	59%	46%	28%	4
Health Navigator (38)	28%	4%	7%	57%	14%	0%	1

## Discussion

In this study, we conducted a qualitative and quantitative analysis of 13 Assessment and Evaluation Tools (AETs) for mental health applications (MH Apps) to identify the strengths and limitations of these tools, understand the existing evaluation criteria, along with assessing their overall quality. We qualitatively analyzed the evaluation criteria of these frameworks which revealed seven key themes: (a) *Authenticity of Content, Source and Process* (b) *Ethical and Legal Issues* (c) *User Experience and User Engagement* (d) *Cost* (e) *Clinical Use and Indications* (f) *Risk to User* (g) *Technology-Related Issues* and (h) *Evidence*. To quantitatively assess the quality, methodological rigor, and transparency of each AET, we used the AGREE II scale (22). We found that: seven AETs met the criteria on the first domain, *Scope and Purpose* (15, 21, 22, 29, 30, 36, 37); three AETs met the criteria on the domain *Stakeholder Involvement* (21, 30, 37); four tools met the criteria for *Rigor of Development* (21, 30, 32, 37); seven AETs met the criteria for *Clarity of Presentation* (22, 29, 33–35, 37, 38); and none of the tools met the criteria for *Applicability or Editorial Independence*. When looking at the AETs overall, only three AETs met the criteria for ‘recommended’ to be used (21, 30, 37), nine were within the ‘weakly recommended’ category (15, 22, 29, 32–36, 38) and one met the criteria for ‘not recommended’ (31).

We found that there is a vast diversity in the terminology used of the AETs, as reported elsewhere (48). This lack of agreement may reflect a lack of consensus among IT professionals (48), which our review supports. Our qualitative analysis of evaluation criteria in AETs led to seven significant IT-related themes, with a lesser focus on clinical topics. While a few AETs mentioned clinical indicators and scrutinized clinical content, the emphasis did not reflect the importance of these areas. The content (i.e., clearly described theoretical background of interventions and assessments) is the primary factor distinguishing one MH App from another.

AETs, in general, did not evaluate *digital literacy* and *access to technology* in their app selection processes. Adequately addressing the *digital divide* is essential for broader implementation and system uptake of MH Apps and AETs. Evaluations of Apps with different, underserved demographic groups with diverse social determinants are needed. It is therefore not surprising that implementation remains the major problem with most AETs. Most AETs do not provide details on how to use the evaluation system and by whom. Without national policies, app developers are regulated by the app distributors such as Google and Apple (and their respective app stores). There is a noticeable absence of app distributors, app developers, health educators, and funders in developing AETs.

Similarly, significant variation exists in how AETs are developed and reported and their use of selection criteria. Most AETs lack rigor in development, and little information is made available on their evaluation and implementation, especially at the broader national health system level. Therefore, most of the AETs reviewed did not meet the criteria for recommendation when their overall quality was assessed using a rating tool (i.e., AGREE II). For example, some AETs consider the app developer or funder’s characteristics, privacy policies, app features, performance characteristics, and ongoing maintenance or updating requirements, while others do not. Other areas of concern include a broad range in purpose and focus of AETs, limited information on stakeholder engagement during AET development, and exclusion or limited inclusion of equity-related issues such as gender, ethnicity, life span, and culture in selection criteria. Many AETs do not consider national or international policies, the resources available and context of health systems. The alignment of international evaluation standards would allow us to compare results across countries and create synergistic international collaborations.

The rapid proliferation of MH Apps has also led to concerns about their use by vulnerable populations. The limited evidence base and the high variance of app quality (including safety concerns) require a consistent and transparent approach when assessing and evaluating their quality. Several forms of AETs, including frameworks, rating scales, and app rating websites, have been published to help raise app quality standards. While some agreement on the technical criteria is considered, these approaches also have significant differences. The aims, scope, purpose, target audiences, and assessment methods vary considerably among these tools. These early efforts are commendable and have paved the path for further developments in this area. However, there is considerable potential for improvement and a need for constant updates to the AETs to reflect the field’s rapid changes. Evaluations also need to be done regularly with the new versions of the App to ensure that quality and safety are guaranteed in all subsequent versions of the App.

The field of AETs for MH Apps is full of complexities. For example, the NHS *Apps Library*, with Apps assessed against a defined set of criteria, was released but quickly rolled back due to public outcry following research that showed privacy and security gaps in a large proportion of the included Apps (49). Furthermore, it has been observed that every 2.9 days, a clinically relevant app for people living with depression becomes unavailable and deleted from app stores (50). Similarly, app stores require regular updates, making it challenging to keep track of a quickly evolving field (51). Many AETs rely upon expert consensus, which can be opaque and difficult to understand for both users and clinicians (42). There is also significant inconsistency in their outcomes. For example, a study of three different ranking systems (PsyberGuide, ORCHA, and MindTools.io) demonstrated a lack of correspondence in evaluating top Apps, indicating weak reliability (10). Evaluations need to show which version of the App was used and what evaluation methods were used. Further work needs to be done to replicate evaluation studies to ensure consistent results in the evaluations.

Tools to assess and evaluate MH Apps are intended to protect the consumer and benefit the creator(s) with guidelines to drive innovation and industry standards. Evaluations must be conducted with the intended users using clear, transparent, and reliable evaluation criteria. Guidelines for reliable evaluation methods need to be developed and more widely used.

Furthermore, there is a lack of interoperability between MH Apps, AETs, and healthcare providers. This could provide an enriching opportunity for continuous improvement of MH Apps and their evaluation based on data entry and engagement with healthcare teams. As such, we found that AETs do not consider culture, ethnicity, gender, language, and life span issues. Current research methods might not be able to address complexities in the field. Most RCTs reporting mHealth Apps do not provide details of the intervention, making the job of AET developers and assessors difficult. Replicability is the litmus test of science, and there is a need to update trial-reporting guidelines to consider these concerns. There is also a general lack of agreement surrounding terminology and definitions of assessment criteria that may have led to misinterpretations for qualitative purposes, even though expert opinion was sought. The replication of studies will create a deeper understanding of how the App performs with different users in diverse geographical regions.

When developed, evaluated and implemented using standardized guidelines, mental health applications (MH Apps) can play an essential part in the future of mental health care (5), making mental health support more accessible and reduce barriers to help-seeking (52). Innovative solutions to the self-management of mental health problems are particularly valuable, given that only a small fraction of people suffering from mood or anxiety problems seek help (53), and even when they want to seek help, support is not always easily accessible (54). Nonetheless, if MH Apps are not well-designed and the App developers do not consider the needs of consumers, MH Apps will not meet the intended expectations. One study of app user engagement of MH Apps reported that the medians of 15-day and 30-day retention rates for Apps were 3.9 and 3.3%, respectively (55). Evaluations of mobile MH Apps that do not have consistent usage and those with low engagement rates cannot be reliably evaluated for efficacy. It is, therefore, crucial to develop research methods that consider these low usage rates, because current methods like RCTs may accurately evaluate these applications in a way that reflects their overall quality. There is also an urgent need to develop guidelines for the clinicians who want to suggest an App or the end users who want to use an App.

The limitations of this study included our search strategy, which was constrained by time and resources available. For this reason, we did not use a comprehensive systematic approach in our search for AETs, which may have led to certain evaluation frameworks being missed. However, one of the strengths of this project was our consultation with stakeholders, including experts in the field of mHealth and MH Apps, that we included to ensure we did not miss any notable AETs. The mixed-methods nature of this project lent itself to a detailed qualitative and quantitative assessment of existing AETs for MH Apps. We used the qualitative approach to identify strengths and limitations of existing AETs and their evaluation criteria, coupled with a quantitative assessment of the quality of AETs and whether or not they were recommended by using a standardized, pre-existing tool (the AGREE II). This is the first project, to our knowledge, that has assessed frameworks for evaluating MH Apps.

## Conclusion

A variety of Assessment and Evaluation Tools (AETs) have been developed to guide users of mental health applications (MH Apps). However, most of these AETs are not very specific to MH Apps and can be used to assess most health Apps. Notably, our qualitative analysis revealed that a limited number of AETs: included MH App content as a criterion for evaluation; discussed the need to consider user characteristics for personalization of use and diversity; considered the use of evidence-base or cost-effectiveness as a criterion; included information on clinical safety; or addressed issues of accessibility, including platform interoperability and users’ digital literacy. Using the AGREE II criteria for overall assessment and recommendations, only three out of 13 AETs we reviewed met the criteria for ‘recommended’, whereas one met the criteria for ‘not recommended’, and the remaining AETs were all within the ‘weakly recommended’ category. There is also minimal agreed-upon terminology in this field, and the AETs reviewing generally lacked focus on clinical issues, equity-related issues and scientific evidence.

Future development of AETs should include criteria that assess cultural acceptability, gender and ethnic/racial diversity, language and lifespan of MH Apps. Additionally, AETs should focus on scientific evidence to assess the effectiveness of an App in a standardized manner. AETs should also strive to reach a consensus surrounding terminology and definitions of assessment criteria to allow for ease of understanding across various MH App users. Importantly, interoperability, especially with healthcare providers, should be a focus of future AETs, to evaluate the technical aspects of data sharing required to improve the coordination of the care continuum and provide more sustainable, effective support for users.

With standardized development, evaluation and implementation guidelines, MH Apps can play an essential role in managing mental health concerns. In order to address stakeholder concerns, AETs should be developed within current laws and government health policies and be supported by evidence-based research methodology, medical education and public awareness. Without continuous and rigorous evaluation, MH Apps will not meet expectations or achieve their full potential to support individuals who need accessible mental health care.

## Data availability statement

The original contributions presented in the study are included in the article/Supplementary material, further inquiries can be directed to the corresponding author.

## Author contributions

All the authors were involved in planning, writing up the application, execution of project and the write up. In addition, specific expertise involved SA being responsible for managing the project along with CT. CT and WK were involved in data collection, analysis and write up. AT and BA went through several drafts and were also involved in knowledge exchange activities. TR is a librarian and carried out library searches. KK, SW, and MA from the MHCC were involved throughout the project in its execution and implementation as well as the knowledge translation activities. KM, MOH, and MIH provided technical expertise in research methods. YQ provided guidance in IT-related issues. FN supervised every stage of the project and was the Principal Investigator. All authors contributed to the article and approved the submitted version.

## Footnotes

^1^ https://Apps.Digitalpsych.Org/

## Supplementary material

### SUPPLEMENTARY DATA SHEET 1

Rating scale AGREE II. Provides the rating scale used to evaluate AETs. Details provided on each domain and the associated items, with a 7-point likert scale ranging from 1 (Strongly Disagree) to 7 (Strongly Agree) to determine how well information meets standards of the item in question.

### SUPPLEMENTARY TABLE 1

AGREE II domain checklist & scoring. Provides overview and description of AETs included. Qualitative analysis of themes and scoring breakdown of each AET on the AGREE II domains, and the overall and domain scoring for each AET is included.
