# Supplementary material for: A mixed methods analysis of existing assessment and evaluation tools (AETs) for mental health applications
Source: Front Public Health. 2024 May 7;12:1196491. doi: 10.3389/fpubh.2024.1196491 (PMC11106355; doi:10.3389/fpubh.2024.1196491)
Supplement: Supplementary file 1 [file Data_Sheet_1.PDF]

Appraisal of Guidelines for Research and Evaluation II

RATING SCALE:

1 Strongly Disagree	2	3	4	5	6	7 Strongly Agree

Strongly Disagree: No information that follows the AGREE II Item.
Strongly Agree: All information exceptionally meets the full standards of the AGREE II Item.

AGREE II ITEM	RATING
**DOMAIN 1. SCOPE AND PURPOSE**	
1. The overall objective(s) of the guideline is (are) specifically described.	
2. The health question(s) by the guideline is (are) specifically described.	
3. The population (patients, public, etc.) to whom the guideline is meant to apply is specifically described.	
**DOMAIN 2. STAKEHOLDER INVOLVEMENT**	
4.The guideline development group includes individuals from all relevant professional groups.	
5. The views and preferences of the target population (patients, public, etc.) have been sought.	
6.The target users of the guideline are clearly defined.	
**DOMAIN 3. RIGOUR OF DEVELOPMENT**	
7. Systematic methods were used to search for evidence.	
8. The criteria for selecting the evidence are clearly described.	
9. The strengths and limitations of the body of evidence are clearly described.	
10. The methods for formulating the recommendations are clearly described.	
11. The health benefits, side effects, and risks have been considered in formulating the recommendations.	
12. There is an explicit link between the recommendations and the supporting evidence.	
13. The guideline has been externally reviewed by experts prior to its publication.	
14. A procedure for updating the guideline is provided.	
**DOMAIN 4. CLARITY OF PRESENTATION**	
15. The recommendations are specific and unambiguous.	
16. The different options for management of the condition or health issue are clearly presented.	
17. Key recommendations are easily identifiable.	
**DOMAIN 5. APPLICABILITY**	
18. The guideline describes facilitators and barriers to its application.	
19. The guideline provides advice and/ or tools on how the recommendations can be put into practice.	
20. The potential resource implications of applying the recommendations have been considered.	
21. The guideline presents monitoring and/ or auditing criteria.	
**DOMAIN 6. EDITORIAL INDEPENDENCE**	
22. The views of the funding body have not influenced the content of the guideline.	
23. Competing interests of guideline development group members have been recorded and addressed.	
